# Do senescence markers correlate in vitro and in situ within individual human donors?

**DOI:** 10.18632/aging.101389

**Published:** 2018-02-28

**Authors:** Mariëtte E.C. Waaijer, David A. Gunn, Diana van Heemst, P. Eline Slagboom, John M. Sedivy, Roeland W. Dirks, Hans J. Tanke, Rudi G.J. Westendorp, Andrea B. Maier

**Affiliations:** ^1^Department of Gerontology and Geriatrics, Leiden University Medical Center, 2300 RC Leiden, the Netherlands; ^2^Unilever Discover, Colworth Science Park, Sharnbrook, Bedfordshire MK44 1LQ, UK; ^3^Netherlands Consortium for Healthy Aging, Leiden University Medical Center, 2300 RC Leiden, The Netherlands; ^4^Department of Molecular Epidemiology, Leiden University Medical Center, 2300 RC Leiden, The Netherlands; ^5^Department of Molecular Biology, Cell Biology and Biochemistry, Brown University, Providence, RI 02912, USA; ^6^Department of Molecular Cell Biology, Leiden University Medical Center, 2300 RC Leiden, The Netherlands; ^7^Department of Public Health and Center for Healthy Aging, Faculty of Health and Medical Sciences, University of Copenhagen, 1123 Copenhagen, Denmark; ^8^MOVE Research Institute Amsterdam, Department of Human Movement Sciences, VU University of Amsterdam, 1007 MB Amsterdam, The Netherlands; ^9^Department of Medicine and Aged Care, Royal Melbourne Hospital, University of Melbourne, Parkville VIC 3050, Australia

**Keywords:** cellular senescence, correlation, markers, *in vitro*, *in situ*

## Abstract

Little is known on how well senescence markers *in vitro* and *in situ* correlate within individual donors. We studied correlations between the same and different *in vitro* markers. Furthermore, we tested correlations between *in vitro* markers with *in situ* p16INK4a positivity.

From 100 donors (20-91 years), cultured dermal fibroblasts were assessed for reactive oxygen species (ROS), telomere-associated foci (TAF), p16INK4a and senescence-associated β-gal (SAβ-gal), with/ without 0.6 µM rotenone for 3 days (short-term). In fibroblasts from 40 donors, telomere shortening, ROS and SAβ-gal were additionally assessed, with/ without 20 nM rotenone for 7 weeks (long-term). In skin from 52 donors, the number of p16INK4a positive dermal cells was assessed *in situ*.

More than half of the correlations of the same senescence markers *in vitro* between duplicate experiments and between short-term versus long-term experiments were significant. Half of the different senescence marker correlations were significant within the short-term and within the long-term experiments. The different senescence markers *in vitro* were not significantly correlated intra-individually with *in situ* p16INK4a positivity. In conclusion, the same and different senescence markers are frequently correlated significantly within and between *in vitro* experiments, but *in vitro* senescence markers are not correlated with p16INK4a positivity *in situ*.

## Introduction

Five decades ago, Hayflick and Moorhead first described the phenomenon of limited replicative capacity of cultured primary cells, termed cellular replicative senescence [1,2]. It was postulated that this *in vitro* (i.e. in cultured cells) phenomenon of stable cell cycle arrest might be related to aging of the whole organisms *in vivo* (i.e. in living organisms). Since then many studies have focussed on cellular senescence *in vitro*, and have identified several triggers inducing senescence as well as pathways leading to senescence (reviewed in [3]). Considerable interest has also been given to the possible *in vivo* implications of senescence; by studying relevant functions, including embryonic development and attenuating liver fibrosis as well as consequences of senescence in animal models, notably age-related diseases, and tumorigenesis [4–8]. In the last few decades [9] tissues have been studied to detect cellular senescence *in situ* (i.e. in tissue), providing knowledge on the prevalence of senescent cells in humans at older ages or with disease.

Apart from growth arrest, several other markers of cellular senescence have been studied (reviewed in [10]). A frequently used marker is senescence-associated β- galactosidase (SAβ-gal) activity, which is upregulated in, but not essential for senescence [9,11]. Other markers are based on triggers of senescence such as DNA damage foci or reactive oxygen species (ROS), expression of genes involved in cell cycle arrest or factors that are secreted by senescent cells [3,10,12]. Most of these markers have been established by detecting senescence *in vitro*, but some can also be used *in situ* [13]. However, the number of studies on fibroblasts reporting on senescence *in situ* compared to *in vitro* is disproportionally small [14], and there is a lack of knowledge concerning the correlation of senescence markers between these conditions. In addition, only a few attempts have been made to study the correlation between different senescence markers.  Our aim is to study the correlations between the same senescence markers (A) and between different senescence markers within individual donors (B), using a unique dataset of highly standardized experiments. These experiments included *in vitro* short-term experiments (1); *in vitro* long-term experiments (2), and *in situ* experiments within skin biopsies (3). First we investigated correlations between the same senescence markers: *in vitro* between duplicate experiments (1A) and *in vitro* between short-term and long-term experiments (2A). In addition, we investigated correlations between different senescence markers: between *in vitro* markers within the same short-term experiments (1B); between *in vitro* markers within the same long-term experiments (2B); and intra-individually between *in vitro* markers and *in situ* p16INK4a positivity in skin biopsies (3B).

## RESULTS

### Characteristics of donors

Table 1 summarizes the anthropometric and medical characteristics of the donors from whom the skin biopsies were obtained based on age (young, mean 23 years; middle-aged, mean 63 years; old, mean 90 years).

**Table 1:** Characteristics of donors.

	Young		Middle-aged		Old		Subset*
	(N=10)		(N=80)		(N=10)		*(N=52)*
Female, no.(%)	7 (70.0)		40 (50.0)		6 (60.0)		*25 (48.1)*
Age, years	22.8 (1.5)		63.2 (7.3)		90.2 (0.5)		*64.2 (6.9)*
Member of long-lived family	n/a		40 (50.0)		n/a		*26 (50.0)*
Body mass index, kg/m^2^	22.2 (1.8)^a^		26.2 (4.1)^b^		25.4 (3.8)		*25.9 (4.3)*
Co-morbidities							
Cerebrovascular accident	0/10 (0.0)		3/76 (3.9)		2/10 (20.0)		*3/51 (5.9)*
Chronic obstructive pulmonary disease	0/10 (0.0)		3/75 (4.0)		1/10 (10.0)		*1/51 (2.0)*
Diabetes mellitus	0/10 (0.0)		7/74 (9.5)		2/10 (20.0)		*4/51 (7.8)*
Hypertension	0/10 (0.0)		17/76 (22.4)		5/10 (50.0)		*15/52 (28.8)*
Malignancies	0/10 (0.0)		3/72 (4.2)		1/10 (10.0)		*1/50 (2.0)*
Myocardial infarction	0/10 (0.0)		0/75 (0.0)		3/10 (30.0)		*1/52 (1.9)*
Rheumatoid arthritis	0/10 (0.0)		0/76 (0.0)		3/10 (30.0)		*0/52 (0.0)*
Smoking, current	0/10 (0.0)		10/76 (13.2)		1/10 (10.0)		*3/48 (6.3)*

SD: standard deviation. a: N=8, b: N=77. N/a: not applicable. Data are depicted as either mean (SD) or number (%). Diseases and intoxications are given as no./total known (%). * This subset was used for the correlation between in vitro senescence markers versus in situ p16INK4a positive human fibroblasts (3B).

### Correlations between the same senescence markers

First, we studied correlations between the same markers, both in non-stressed and stressed conditions. The correlation of duplicates of each senescence marker (p16INK4a, telomere associated foci - TAF, reactive oxygen species - ROS and senescence-associated β-gal - SAβ-gal) were tested between experiment I and II of the short term experiments (Table 2). Most markers showed a significant association between experiments I and II (coefficients > 0.400), except for ROS which showed low, non-significant correlation coefficients.

**Table 2:** Senescence markers and their correlations between duplicate short-term experiments (1A).

	Distribution of markers			
	Experiment I	Experiment II	Correlation coefficient	P-value
**Non-stressed**				
p16INK4a, %	0.90 (0.45; 1.65)	1.61 (0.76; 2.71)	0.702	<0.001
TAF, %/nucleus	24.2 (16.9; 31.0)	24.4 (18.5; 32.1)	0.418	<0.001
ROS, FI	1477 (1280; 1706)	1455 (1295; 1762)	-0.111	0.354
SAβ-gal, FI	2959 (2389; 3813)	2987 (2187; 3951)	0.527	<0.001
				
**Stressed**				
p16INK4a, %	2.17 (1.10; 4.17)	4.70 (2.33; 6.48)	0.623	<0.001
TAF, %/nucleus	20.6 (14.8; 27.9)	21.9 (16.0; 26.7)	0.414	<0.001
ROS, FI	2003 (1734; 2376)	1972 (1653; 2366)	0.139	0.244
SAβ-gal, FI	4251 (3405; 5345)	4044 (3180; 5233)	0.452	<0.001

N=100. Marker distribution is given as median (IQR). Correlations are Pearson’s partial correlation coefficient, adjusted for batch. All markers in experiment I were correlated with the same markers in experiment II. FI: fluorescence intensity. P16INK4a: percentage of p16INK4a positive cells; TAF (telomere associated foci): percentage of nuclei with ≥1 TAF/nucleus; ROS: mean fluorescence intensity peak reactive oxygen species; SAβ-gal: median fluorescence intensity peak senescence-associated β galactosidase.

Table 3 shows the correlations between ROS and SAβ-gal in the short-term versus the long-term experiments. The mean of ROS measures in the short-term experiment were significantly correlated to ROS in the long-term experiment. SAβ-gal was not significantly correlated between the short-term and long-term experiments.

**Table 3:** Senescence markers and their correlations between short-term versus long-term experiments (2A).

	Distribution of markers			
	Short-term experiment	Long-term experiment	Correlation coefficient	P-value
**Non-stressed**				
ROS, FI	1559 (1356; 1734)	1500 (1366; 2205)	0.419	0.010
SAβ-gal, FI	2973 (2445; 3732)	3452 (2905; 4660)	-0.009	0.959
				
**Stressed**				
ROS, FI	2095 (1753; 2324)	1835 (1553; 2205)	0.426	0.009
SAβ-gal, FI	4171 (3530; 5231)	4090 (3417; 5205)	-0.006	0.972

N=40. Correlations are Pearson’s partial correlation coefficient, adjusted for batch. All mean markers of short-term experiments I and II were correlated with the same markers in the long-term experiment. FI: fluorescence intensity. ROS: mean fluorescence intensity peak reactive oxygen species; SAβ-gal: median fluorescence intensity peak senescence-associated β galactosidase.

**Figure 1:**
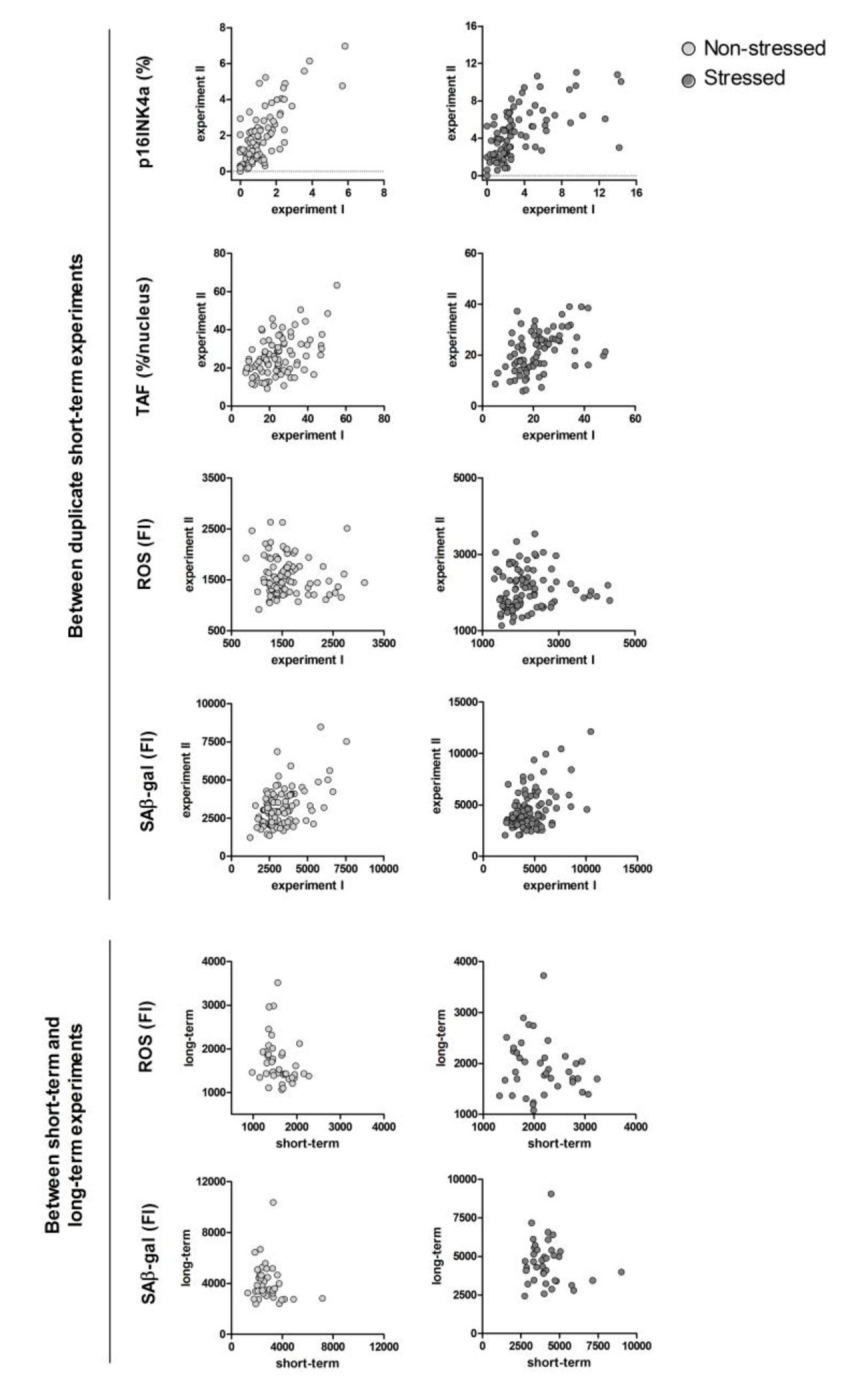
**Correlation plots of the same senescence markers between duplicate experiments and between short-term versus long-term experiments.** Each dot represents an individual donor, N=40-100. Uncorrected (not log transformed) data points are shown. P16INK4a: percentage of p16INK4a positive cells; TAF (telomere associated foci): percentage of nuclei with ≥1 TAF/nucleus; ROS: mean fluorescence intensity peak reactive oxygen species; SAβ-gal: median fluorescence intensity peak senescence-associated β galactosidase. For the between short-term and long-term experiment correlations, in vitro variables are the mean of short-term experiments.

The raw data points of the same markers between duplicate experiments and between short-term and long-term experiments are plotted in Figure 1 for visualisation.

### Correlations between different senescence markers

**Figure 2:**
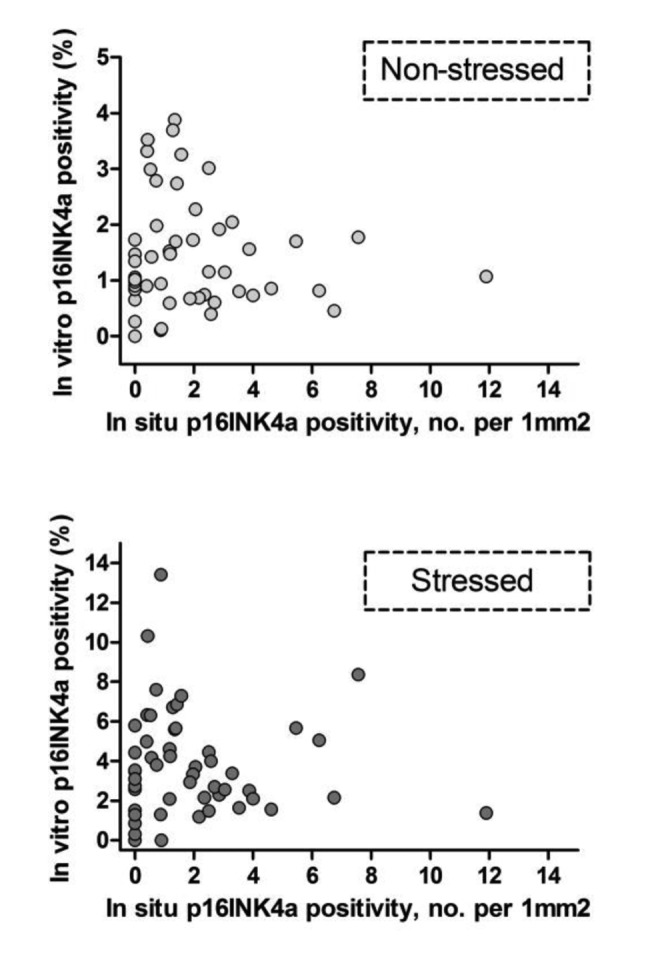
**Intra-individual correlations: *in vitro* versus *in situ* p16INK4a positivity**. Each dot represents an individual donor, N=52. *In vitro* p16INK4a positivity: percentage of p16INK4a positive cells - mean of experiments I and II. *In situ* p16INK4a positivity: number of p16INK4a positive cells per 1mm^2^ dermis. Uncorrected (not log transformed) data points are shown.

Second, we studied correlations between different senescence markers. In the Supplementary Material, correlations between different senescence markers within the short-term (Supplementary Table 1) and long-term experiments (Supplementary Table 2) are given. In the short-term experiment each marker was tested against the 3 other markers, both in non-stressed (6 combinations) and stressed condition (6 combinations). Of these 12 senescence marker combinations, 6 were significantly correlated (in non-stressed and stressed conditions 3 each). P16INK4a showed the highest correlations with other markers. In the long-term experiment a total of 6 marker combinations were tested in both non-stressed and stressed conditions of which 3 senescence marker combinations were significantly correlated, mainly with ROS (2 in the non-stressed condition, 1 in the stressed condition). Using telomere length instead of telomere shortening reduced the amount of significant markers combination correlations, indicating a difference in telomere length and telomere shortening over time as senescence markers. *In vitro* senescence markers (both in non-stressed and stressed conditions) were tested for correlation with *in situ* p16INK4a positivity of dermal fibroblasts (Table 4). No significant correlations were observed between *in situ* p16INK4a positivity and any of the *in vitro* senescence markers (ROS, TAF, SAβ-gal or p16INK4a). In Figure 2, *in vitro* p16INK4a positivity in non-stressed and stressed conditions are plotted against *in situ* p16INK4a positivity of dermal fibroblasts, further showing this lack of intra-individual correlation.

**Table 4:** Intra-individual correlations: *in vitro* senescence markers versus *in situ* p16INK4a positive human fibroblasts (3B).

	Coefficient	P-value
**Non-stressed**		
p16INK4a	0.064	0.655
TAF	-0.030	0.835
ROS	-0.097	0.498
SAβ-gal	-0.042	0.772
		
**Stressed**		
p16INK4a	0.091	0.527
TAF	0.014	0.922
ROS	-0.095	0.506
SAβ-gal	0.023	0.871

Values are depicted as Pearson's partial correlation coefficient, adjusted for batch. Data for *in situ* and *in vitro* senescence markers were available for N=52 donors. P16INK4a positive dermal fibroblasts: number of positive cells per 1mm2 dermis. All *in vitro* variables are the mean of short-term experiments. P16INK4a: % of p16 positive cells; ROS: mean fluorescence intensity peak; SAβ-gal: median fluorescence intensity peak; telomere-associated foci (TAF): % of nuclei with ≥1 53BP1 foci per nucleus, coinciding with telomeric DNA.

## DISCUSSION

In individual donors, half of the correlations of the same senescence markers in vitro were significant between duplicate experiments (1A) and between short-term versus long-term experiments (2A). Within the experiments the different senescence markers were significantly correlated to each other in half of the correlations tested, both in short-term (1B) and long-term experiments (2B). In general, correlation coefficients were lower as compared to those calculated for the same senescent markers. Assessment of correlations between *in situ* p16INK4a positivity with different *in vitro* senescence markers showed a lack of correlation, both with *in vitro* markers in non-stressed and stressed conditions (3B).

Most correlations between duplicate experiments show that the experiments were adequately reproducible, suggesting that the influence of technical issues was limited. However, ROS showed poor reproducibility between duplicates which hampers interpretation of other tested correlations with ROS. The fact that no correlation coefficient above 0.702 was observed indicates that despite highly standardized conditions, the assays used are inherently prone to variation. Although the same markers were also correlated between the short-term and long-term experiments, this was less often the case than for the between duplicate experiment correlations. This finding is not surprising, as cell strains of an individual could respond to short-term and long-term stress differently. In our previous study we showed that SAβ-gal in the stressed condition in the short-term experiment was negatively associated with the maximum replicative capacity of the strain (a long-term outcome), whereas a positive but nonsignificant trend was seen in the non-stressed condition [15].

Senescence can be triggered in response to multiple factors and be induced through different pathways. Therefore it has been advised to use a marker of cell cycle arrest plus a minimum of two senescence markers [16]. Studies on senescence markers in single cells show that there is not a hundred percent concordance of different markers, for e.g. p16 and SAβ-gal [17], p16 and p21 [18], and γH2AX foci and p21 [19]. One of these studies also showed that SAβ-gal, senescence associated heterochromatin foci and the combination of Ki67 with γH2A.X foci were superior to other marker combinations in predicting growth curves of MRC5 fibroblast cultures [19]. A recent review [13] discussed the shortcomings of frequently used markers to assess *in vitro* senescence and particularly the difficulties of using these markers to detect *in vivo* senescence. It is a topic under debate in a still rapidly evolving field. We confirm the importance of this stance based on our results on correlations between different senescence markers. Only a half of the tested senescence marker combinations were significantly correlated within the experiment. The *in vitro* senescence marker that was most correlated to other *in vitro* senescence markers was p16INK4a. This was also the marker with the highest correlation coefficient between experiment I and II (between duplicate experiments). This good correlation of duplicates could thus explain the observation that p16INK4a correlated most frequently to the other markers, and overall appears to be the most robust *in vitro* senescence marker from the set of markers tested here.

A recent review has shown that while some *in vitro* observations on fibroblast aging have also been observed *in situ* in skin tissue, many observations have not been tested *in situ* yet [14]. To our knowledge, this is the first study in humans to directly correlate senescence markers *in vitro* and *in situ* in cultured fibroblasts and biopsies from the same individual to assess whether both are reflective of a common (epi)genetic propensity to induce cellular senescence. In mice microRNA expression profiles were compared in cultured cells and aged mouse brains, which showed only very little similarities in expression [20]. The lack of correlation between *in vitro* and *in situ* senescence markers we have observed, was not altogether surprising. While experimental set-ups allow controlling of many variables, this also decreases the natural context of human cells. It has been observed that the process of establishing fibroblasts strains from skin biopsies itself can result in a selection of a subgroup of fibroblasts. Fibroblasts from subsequent outgrowths of single skin biopsies were shown to differ in their proliferation capacities [21]. Outgrowth from different dermal layers results in higher culture survival time in fibroblasts from the papillary dermis compared to reticular dermis [22,23]. Hence, *in vitro* fibroblasts might only relate to a small sub-population of fibroblasts *in situ* and study of this sub-population *in situ* might be needed to detect any correlations. In addition, different culturing conditions were shown to have effects on replicative lifespan as well [24], and the process of cell culture itself has been suggested to drive some of the senescence findings *in vitro* [25,26]. We used atmospheric oxygen culture conditions which in itself is thought to be a stressor [27]. This can be seen in our scatterplot showing some individuals with high p16INK4a positivity *in situ* and low p16INK4a positivity *in vitro*, which might have resulted from selection of senescence resistant fibroblasts during expansion. Furthermore, a small biopsy from one location of a donor might not reflect the entire tissue or entire organism adequately. It is also not clear how a fluctuating physiological state of a donor can influence the samples tissue. Overall, *in vitro* experimental data from cells derived from one individual might not be representative for the cell populations in their tissues under *in vivo* conditions. Due to this lack of intra-individual correlation, difficulty might arise in extrapolating observations from *in vitro* experiments to *in vivo* implications. On the other hand, perhaps the *in vitro* characteristics of the selected subpopulation of primary cells could still reflect *in vivo* cellular capacities in specific situations, such as disease or in the presence of environmental stressors.

This study uses unique data on multiple senescence markers *in vitro* established from 100 individual fibroblast strains. We regard the high number of fibroblast strains as a strong point of this study. All culturing procedures and experiments were conducted under highly standardized conditions, and our results here highlight the need for maximally standardize the operating procedures. We measured senescence markers in cultures that were in phase IIa, and thus just a fraction of cells were senescent, reflecting the mix of dividing and senescent cells within the human body. Studying correlations in only non-dividing cultures might yield different findings. A limitation of the study is that we did not include a marker for proliferation such as Ki-67. The association between *in vitro* and *in situ* p16INK4a positivity could only be evaluated in 52 middle aged subjects that had both measurements, which limited the power to detect significant correlations. Another limitation of the present study is at the same time a limitation of many human studies in general: we have detected p16INK4a *in situ*, but cannot (yet) study cellular senescence *in vivo* in humans. Studies aiming to detect cellular senescence *in vivo* in animal models have shown high inter-individual variability, especially at older ages [28,29]. Inter-individual variation of senescence might also be influenced by genetic polymorphisms. In human peripheral blood T-cells, one atherosclerotic disease-related SNP was shown to associate with decreased expression of INK4/ARF transcripts [30]. Further analysis of intra-individual correlation between *in vitro* and *in vivo* senescence associated markers within animal models could help to better explore this lack of correlation. Another limitation of our study is that for *in situ* measurements we only have data of one senescence marker, p16INK4a, whilst consensus is lacking on which (panel of) markers should be used to appropriately detect senescent cells *in situ*.

In conclusion, on an individual donor level the same markers of senescence *in vitro*, and to a lesser extent different markers of senescence, are frequently significantly correlated within and between experiments. *In vitro* senescence markers and *in situ* fibroblast p16INK4a positivity were not correlated. Caution is warranted when interpreting results from *in vitro* senescence studies towards *in vivo* implications.

## METHODS

### Study design

The Leiden 85-plus Study is a prospective population-based study [31] in which inhabitants of Leiden (the Netherlands) were invited to participate upon reaching the age of 85 years between 1997 and 1999. Several phenotypes of participants were collected, amongst them information on chronic diseases, disabilities, cognitive function and well-being. In order to study *in vitro* fibroblast characteristics based on large chronological age differences participants aged 90 years from the Leiden 85-plus cohort donated skin biopsies of the upper inner arm together with young voluntary controls aged 18-25 years [32]. As previously described [33], in the Leiden Longevity Study factors contributing to familial longevity are studied. Long-lived siblings (men aged 89 years or over, women age 91 years or over) were included, as well as their offspring who are assumed to have a familial propensity for longevity as well. The partners of these offspring were included in former studies as controls, as they are of similar age and share the same environment. Skin biopsies for *in situ* staining and fibroblast cultures were obtained from middle-aged to old (mean 63 years) offspring of nonagenarian sibling and their partners. All participants in these studies have given written informed consent, and both studies were approved by the Medical Ethical Committee of the Leiden University Medical Center.

### *In vitro* senescence markers

Detailed methods have been described previously [34–36]. In short, fibroblast strains from 10 young donors (passage 14), from 80 middle-aged donors (40 offspring of long-lived families and 40 partners – passage 10), and from 10 old donors (passage 14) were randomly selected for subsequent experiments. Fibroblasts were cultured for 3 days with or without 0.6 µM rotenone (a mitochondrial complex I inhibitor) added to the medium (short-term experiments). Senescence can be triggered by stressors such as ROS-induced damage through rotenone [16,37].

Adding a stressor can show cellular responses to stress-induced damage, in addition to mainly replicative senescence in non-stressed conditions. The following senescence markers were assessed in fibroblast cultures in non-stressed and in rotenone-stressed conditions: β galactosidase (SAβ-gal), reactive oxygen species (ROS), p16INK4a positivity and telomere-associated foci (TAF). Fluorescence intensities were measured using flow cytometry, resulting in median fluorescence intensity values of SAβ-gal and mean fluorescence intensity values of ROS. The percentages of immunocytochemically stained p16INK4a positive fibroblasts were determined. The number of telomere-associated foci (TAF) was determined using immunofluorescence and PNA telomeric probe (53BP1 positive foci located at telomeres). 100 randomly selected nuclei were automatically scored for TAF. TAF are presented as the percentage of nuclei with ≥1 TAF per nucleus. These experiments were conducted in duplicate (experiments I and II) [34,36] (i.e. in parallel conducted repeated experiments for each strain – at passage 14 a donors culture was split and experiment I was performed during 3 days (both stressed and non-stressed condition); after one week the other half of the culture underwent the same procedure, which was experiment II.). Furthermore, alongside the above mentioned experiments, 10 fibroblast strains from young, 20 from middle-aged (10 offspring, 10 partners) and 10 from old donors were randomly selected and cultured for 7 weeks, with or without 20 nM rotenone to generate chronic stress (long-term experiments). The median fluorescence intensity values of β galactosidase (SAβ-gal) and reactive oxygen species (ROS) were measured using flow cytometry. Telomere length was assessed with a flow-FISH kit and was expressed as the percentage compared to the reference cell line. The telomere shortening rate was further determined by comparing these measurements to telomere length at baseline and dividing the difference by the number of cumulative population doublings [35].

### *In situ* senescence marker

As detailed previously [38], in order to detect p16INK4a in the formalin fixed paraffin embedded skin tissue, immunohistochemistry staining was used. Dermal p16INK4a positive cell counts were restricted to morphologically determined fibroblasts and normalized for the area of the dermis in which the cells were counted. Dermal p16INK4a positivity is given as the number of p16INK4a positive cells per 1 mm^2^.

### Statistics

**Figure 3:**
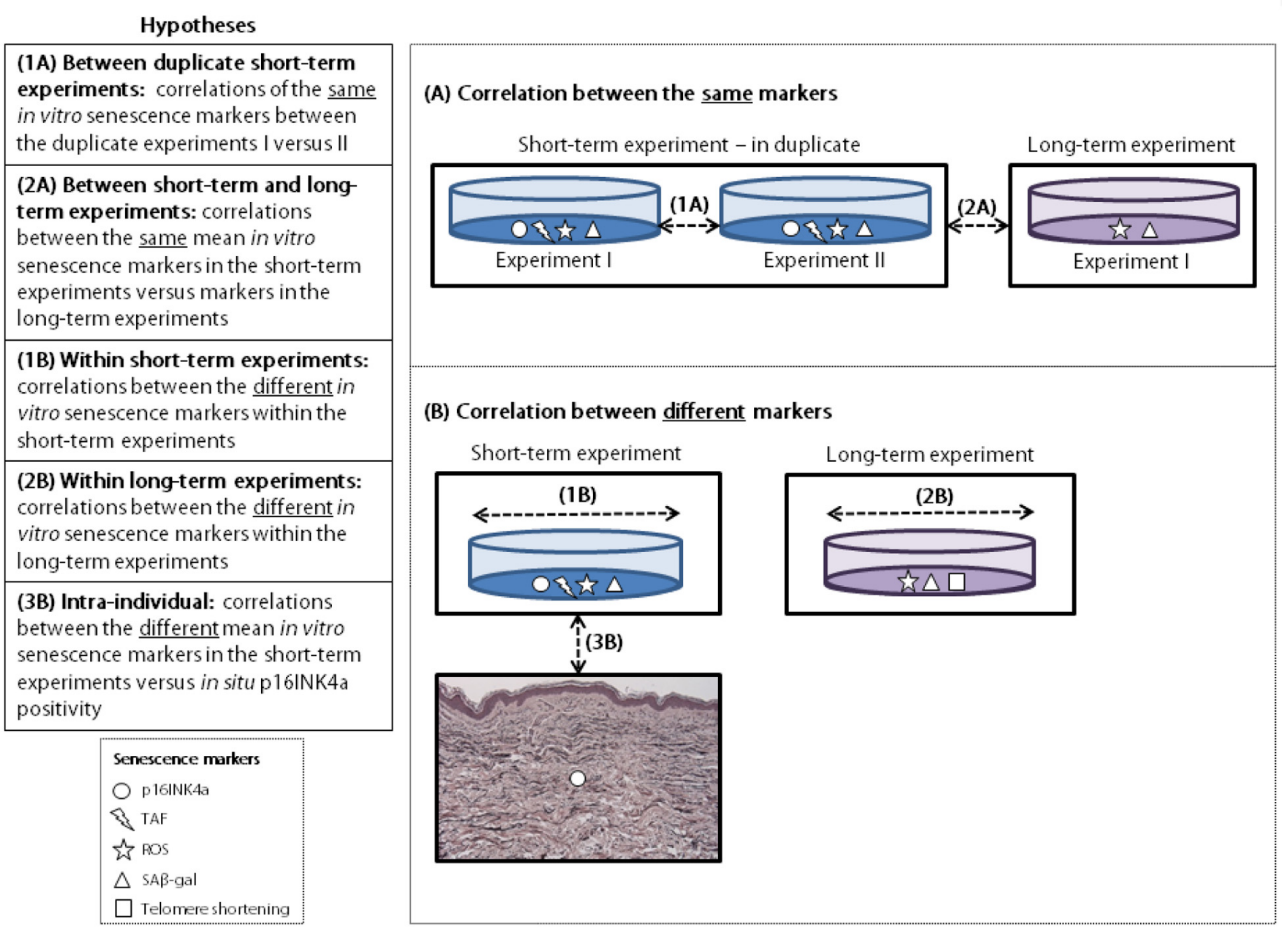
**Explanation of hypotheses tested.** TAF: telomere associated foci. ROS: reactive oxygen species. SAβ-gal: senescence-associated β galactosidase.

All analyses were performed using IBM SPSS Statistics 20. Not all data was normally distributed and these variables were naturally log transformed before evaluating the correlations by calculating the Pearson partial correlation coefficient, adjusted for experiment batch. The studied correlations are explained in Figure3. First, correlations of the same senescence markers were analyzed using data of the short-term experiments I and II (duplicate experiments) (1A); the mean results of duplicates in the short-term experiments and the single measurements of the long-term experiments (as this experiment was performed once) (2A). Secondly, correlations between different senescence markers were analyzed using the mean results of duplicates within the short-term experiments (1B); the single measurements within the long-term experiment (2B); and the mean of the *in vitro* markers in the short-term experiments (mean results of duplicates) and *in situ* p16INK4a positivity (3B). For the latter correlation a subset of N=52 donors was used that had senescence markers measured both in their cultured fibroblasts (*in vitro*) and in their skin biopsies (*in situ*). All *in vitro* markers were measured in a non-stressed and (rotenone) stressed condition. For data visualization the percentage of fibroblasts staining positive for p16INK4a *in vitro* was plotted against the number of p16INK4a positive dermal cells *in situ* using Prism Graphpad 5 software.

## Supplementary Material

Supplementary File
