# Supplementary material for: Do senescence markers correlate in vitro and in situ within individual human donors?
Source: Aging (Albany NY). 2018 Feb 28;10(2):278–89. doi: 10.18632/aging.101389 (PMC5842854; doi:10.18632/aging.101389)
Supplement: Supplementary File [file aging-10-101389-s001.pdf]

SUPPLEMENTARY MATERIAL

Supplementary Table 1. Correlations between the different *in vitro* markers within the short-term experiment (1B).

	p16INK4a	TAF	ROS	SAβ-gal
**Non-stressed**				
p16INK4a	n/a	-	-	-
TAF	**0.253 (0.011)**	n/a	-	-
ROS	**0.321 (0.001)**	-0.048 (0.636)	n/a	-
SAβ-gal	0.151 (0.134)	0.054 (0.598)	**0.232 (0.021)**	n/a
**Stressed**				
p16INK4a	n/a	-	-	-
TAF	**0.227 (0.024)**	n/a	-	-
ROS	**0.299 (0.003)**	0.038 (0.706)	n/a	-
SAβ-gal	0.162 (0.109)	**0.215 (0.032)**	0.115 (0.255)	n/a

N=100. Values are depicted as Pearson's correlation coefficient (P-value), partial correlation with adjustment for batch. n/a: not applicable. P16INK4a: % of p16 positive cells; Telomere-associated foci (TAF): % of nuclei with ≥1 53BP1 foci coinciding with telomeric DNA per nucleus; ROS: mean fluorescence intensity peak; SAβ-gal: median fluorescence intensity peak. All *in vitro* variables are the mean of duplicate experiments.

Supplementary Table 2. Correlations between the different *in vitro* markers within the long-term experiment (2B).

	ROS	SAβ-gal	Telomere shortening	*Telomere length*
**Non-stressed**				
ROS	n/a	-	-	-
SAβ-gal	**0.341 (0.042)**	n/a	-	-
Telomere shortening	**-0.466 (0.004)**	-0.011 (0.949)	n/a	*n/a*
Telomere length	*-0.100 (0.550)*	*0.280 (0.093)*	*n/a*	*n/a*
**Stressed**				
ROS	n/a	-	-	-
SAβ-gal	**0.436 (0.008)**	n/a	-	-
Telomere shortening	-0.220 (0.198)	-0.030 (0.862)	n/a	n/a
Telomere length	*0.156 (0.358)*	*0.276 (0.104)*	*n/a*	*n/a*

N=40. Values are depicted as Pearson's correlation coefficient (P-value), partial correlation with adjustment for batch. n/a: not applicable. ROS: mean fluorescence intensity peak; SAβ-gal: median fluorescence intensity peak; Telomere shortening: percentage of shortening per population doubling; Telomere length: the percentage compared to the reference cell line
